# Editorial: Go with the vet-flow! The current uses and new frontiers of flow cytometry in veterinary sciences, volume II

**DOI:** 10.3389/fvets.2026.1793232

**Published:** 2026-02-09

**Authors:** Fulvio Riondato

**Affiliations:** Department of Veterinary Sciences, School of Agriculture and Veterinary Medicine, University of Turin, Torino, TO, Italy

**Keywords:** conventional and spectral flow cytometry, food safety, immunophenotyping, oncology, platelet, veterinary

Publications in veterinary journals employing flow cytometry have increased markedly over the past 6 years, reflecting the growing integration of this technology into veterinary research and diagnostics. In 2024, we launched the Research Topic “*Go with the vet-flow! The current uses and new frontiers of flow cytometry in veterinary sciences”* to provide an overview of current applications and, for the first time, create a dedicated space within the journal for colleagues working in this rapidly evolving field. This initiative was very well-received and resulted in the publication of 12 articles spanning a wide range of animal species, biological samples, and analytical targets. Given the continued growth of flow cytometry and its methodological innovations, and in recognition that a single volume could not offer an exhaustive overview, we expanded the initiative to include a second volume.

This second volume comprises six additional contributions that further illustrate the breadth and maturity of flow cytometry applications in veterinary medicine, predominantly in a clinical context. The articles range from the development and validation of analytical strategies to the characterization of reference populations in healthy and diseased animals and the identification of novel cell populations and biomarkers. Collectively, these studies provide a robust foundation for more refined phenotypic characterization and future assessment of prognostic significance in prospective clinical investigations.

Five of the six articles focus on dogs or cats, and three address oncological conditions. Iamone et al. address a fundamental and frequently unmet need in clinical diagnostics by providing a rigorous evaluation of the analytical performance of a cytometric panel for the quantification of mast cells in canine peripheral blood, bone marrow, and lymph node aspirates. Their study demonstrates the reliability of flow cytometry for identifying and quantifying mast cells in all three matrices, supporting the validity of previously proposed diagnostic and prognostic cut-offs in dogs with mast cell tumors (1, 2). Meneses-Nava et al. characterize the immunophenotypic profiles of circulating lymphocytes in healthy cats, cats without hematopoietic neoplasms, and cats with chronic lymphocytic leukemia using a 10-marker panel. Three principal subsets are identified according to CD5, CD21, and CD45R expression, and the expression of CD4, CD8, MHC class II, CD134, and CD9 is also described. The inclusion of forward scatter and mean fluorescence intensity data provides valuable quantitative reference information. Although preliminary, this study addresses a significant knowledge gap in feline immunophenotyping and offers an important framework for diagnostic interpretation. Blockeel et al. explore the use of an anti-CD94 antibody clone for the immunophenotyping of lymphocytes in canine blood and lymph nodes. The authors report CD94 expression in healthy dogs and in dogs with leukemia or lymphoma, identifying phenotypic profiles consistent with natural killer (NK) cells and NK-like T cells, in addition to noting marked interindividual variability. These exploratory findings provide a basis for future investigations into the role of CD94-positive cells within the tumor microenvironment and the prognostic and therapeutic relevance of CD94 expression in canine neoplasia, with potential comparative significance given the growing interest in this target in human oncology (3).

Two further contributions focus on non-oncological clinical applications. McDonald et al. characterize circulating leukocyte populations in dogs and cats with pruritus and in control animals by combining spectral cytometry with unsupervised, machine learning–based clustering. This analytical strategy enables the identification of rare or previously unrecognized cell populations that would likely remain undetected using conventional manual gating approaches. The study illustrates the feasibility and added value of advanced computational pipelines in veterinary cytometry, even in the context of current limitations in marker availability and panel complexity. Shaverdian et al. present a standardized method for the characterization of procoagulant platelets in cats based on the assessment of mitochondrial membrane potential, phosphatidylserine exposure, and P-selectin expression. The authors provide detailed methodological guidance and introduce a scoring system to estimate the procoagulant tendency of individual subjects, thereby proposing a potential biomarker for the assessment of thromboembolic risk in feline patients.

Although flow cytometry is primarily used in veterinary medicine, as in human medicine, to characterize immune cell populations, its scope extends to additional domains of relevance. Of particular importance from a One Health perspective is food safety, where flow cytometry offers a promising alternative to conventional reference methods for the detection of hazardous or prohibited substances (4). Cannizzo et al. exemplify this potential by applying a commercially available multiplex assay for the detection of antibiotic residues in chicken muscle and by highlighting the suitability of flow cytometry as an efficient screening tool for monitoring compliance with regulatory limits.

Taken together, the studies included in the first and second volumes of this Research Topic illustrate both the vitality and the rapid methodological evolution of flow cytometry in veterinary science. However, expertise remains fragmented across different domains and areas of research. The continued expansion of this discipline and its technological sophistication underscore the need for stronger interaction among specialists, the consolidation and recognition of specific competencies, and the establishment of dedicated platforms for discussion, training, and dissemination. This Research Topic represents an initial step toward building a cohesive veterinary cytometry community. The positive reception of the first two volumes and the strong participation in the meeting “Vets flowing on—First European workshop on veterinary and life sciences flow cytometry” held in Turin, Italy on 21 November 2025, indicate a growing interest in this field and suggest that the time is right to promote flow cytometry as an autonomous and recognized discipline within veterinary medicine. We therefore encourage all colleagues working in veterinary cytometry to actively seek collaboration, engage with the broader cytometry community, and contribute to the development of a shared scientific identity.
